# Illegal trade of songbirds: an analysis of the activity in an area of northeast Brazil

**DOI:** 10.1186/s13002-020-00365-5

**Published:** 2020-03-30

**Authors:** Wallisson Sylas Luna de Oliveira, Anna Karolina Martins Borges, Sérgio de Faria Lopes, Alexandre Vasconcellos, Rômulo Romeu Nóbrega Alves

**Affiliations:** 1 Programa de Pós-Graduação em Ciências Biológicas (Zoologia), Departamento de Sistemática e Ecologia, Universidade Federal da Paraíba, Via Expressa Padre Zé, s/n, Cidade Universitária, João Pessoa, PB 58059-970 Brazil; 2 Departamento de Biologia, Universidade Estadual da Paraíba, Av. Baraúnas, 351, Campus Universitário I, Bodocongó, Campina Grande, PB 58109-753 Brazil

**Keywords:** Wild birds, Ethnozoology, Biodiversity conservation, Song attractiveness

## Abstract

**Background:**

This study aimed to analyze the chain and dynamics of the trade of wild birds between keepers and traders in an area of northeast Brazil. Profit from the purchase and sale of these animals in the trade chain was also estimated.

**Methods:**

The information was obtained through interviews with direct participants in the wild bird trade chain.

**Results:**

We recorded a total of 34 bird species involved in illegal trade. In general, the purchase and sale values of songbirds are associated with the attractiveness and songs of the birds. Regarding the commercial potential of the species, those with high numbers of traded individuals had higher average purchase values and, especially, sale values. Birds with lower purchase values showed higher sale profits and were sold in large numbers. The purchase and sale values of songbirds in the present study show a significant economic return for those involved in this activity.

**Conclusions:**

The results of this study may provide data to support future studies on the conservation of wild birds, assisting in monitoring illegal trade, a persistent problem in the region studied.

## Background

As the third largest illegal activity in the world, the illegal trade of wild animals is only surpassed by the trafficking of drugs and arms, constituting an activity that generates billions of dollars annually [1, 2] and affects one third of bird species worldwide, followed by thousands of reptile, amphibian, mammal, and fish species [3–11].

In Brazil, the greatest threat to wildlife after habitat loss and subsistence hunting is illegal trade [12–14], which has directly impacted biodiversity, with some populations already near extinction [15]. It is estimated that in Brazil, 38 million specimens are captured from the wild every year, of which 4 million are used for illegal trade [16], and birds are the group of animals most important in the trafficking of wild animals, accounting for approximately 80% of the total number of wild animals illegally traded [3, 17].

Highly desirable due to the beauty of their plumage and song attractiveness, Passeriformes are a resource of considerable economic value in various regions of Brazil, especially in the Northeast Region. Little is known about the trade of these birds at the national level [18], perhaps because it is an illegal activity, which makes it difficult to obtain reliable data about this activity. According to Alves et al. [3], once captured, birds are sold at low prices in their rural areas and then resold in small towns or transported for sale in large urban centers. Many studies on the use and trade of pet birds, especially in the Northeast Region of Brazil, have been published in recent years [19–28], but studies that provide more detailed information on trade routes and dynamics, including the prices of the species involved and profitability, are still scarce.

Therefore, this study aims to analyze the chain and dynamics of the trade of wild birds between local respondents and other traders from areas of the semiarid region of northeast Brazil, a region where illegal trade is widespread. We use an ethnozoological approach to estimate the profit from the purchase and sale of these animals in the local trade chain. Thus, we seek to answer the following questions: What are the social factors that influence the trade of songbirds in the region studied? What are the characteristics of the chain and dynamics of the trade of these animals? What species of birds are traded and what criteria are adopted to choose these animals?

## Methods

### Study area

**Fig. 1:**
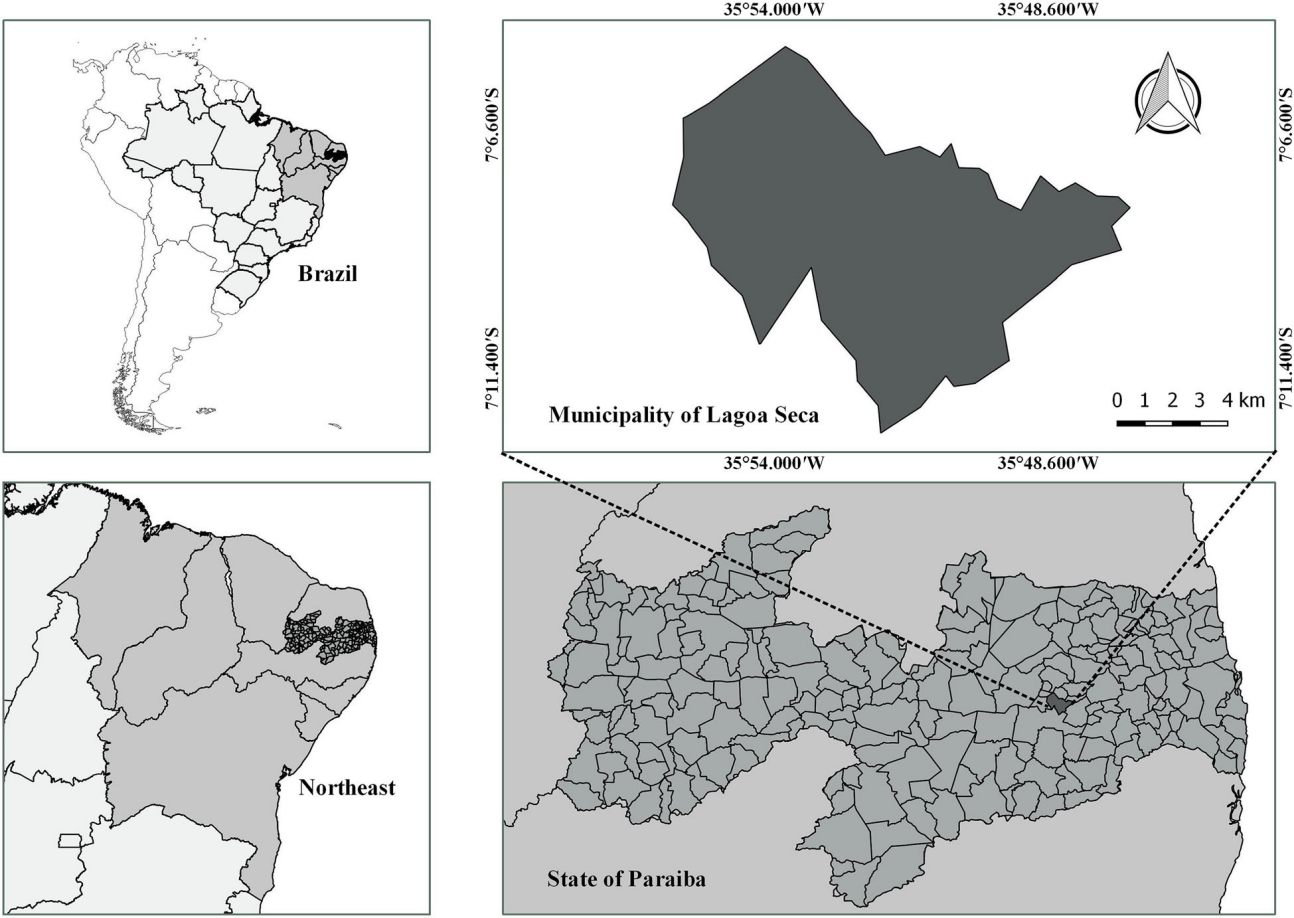
Location of the Lagoa Seca municipality, Paraíba Agreste mesoregion, Brazil

The study was conducted in the Lagoa Seca municipality located in the state of Paraíba (Fig. 1), Northeast Region of Brazil. Lagoa Seca is located in the Paraíba Agreste mesoregion and part of the Borborema Plateau geoenvironmental unit and is located 109.4 km from the state capital, João Pessoa. With an area of 107.589 km^2^, the Lagoa Seca municipality has a population of 25,900 inhabitants, with 10,570 urban inhabitants and 15,330 rural inhabitants [29]. Its Human Development Index (HDI) is 0.627, according to the Human Development Atlas [30]. The vegetation of this unit consists of tropical dry forests [31], and the climate is rainy tropical hot and humid—class A, AS’ (Köppen classification). The main economic activities of the municipality are trade and agriculture.

### Procedures

#### Study approval and data collection

The study was approved by the Research Ethics Committee of Lauro Wanderley University Hospital (HULW) under CAAE no. 50219015.2.0000.5183.

Bird keepers and traders from the urban area and rural communities of the Lagoa Seca municipality were opportunistically visited between the months of October 2015 and March 2017 for collecting information. Initially, informal conversations with the first interviewees took place to gain their trust. During those conversations, the nature and objectives of the study were explained, and the interviewees were asked for their permission to record the information. Later, data on the use of birds were collected through semi-structured interviews complemented with unstructured interviews and informal conversations [32, 33]. The applied forms included information on socioeconomic aspects (income, education level, occupation, and housing), frequency and motives of bird use, abundance of species in the region (very low, low, medium, and high), and commercial information.

The forms also contained questions regarding the training and maintenance of birds in captivity (breeding period, estimated costs, and characteristics of the song of the animal) and the best period of the year to capture them. Based on the first interviews performed, the informants were selected using the “snowball” technique [34], which consists of using information provided by the interviewee to the researcher to recruit another informant.

Much of the information was obtained from direct observations [35] during the monitoring of the capture, maintenance, purchase and sale of wild birds among the informants, and visits to the street markets where these birds are traded in the region.

#### Identification of species

The identification of the cited birds occurred in the following ways: (1) direct observation in the house of the interviewees and in the street markets; (2) photographic records during the interviews; (3) the use of the checklist interview technique [36], in which photographs of birds are shown to the interviewees; (4) identification by taxonomists familiar with the avifauna of the study site; and (5) based on ethnoornithological studies previously performed in the northeast semiarid region [20, 25, 27, 37].

After species identification, the scientific nomenclature used followed the guidelines of the Brazilian Ornithological Records Committee [38]. The conservation status of the species recorded was determined based on the Brazilian List of Endangered Species [39] and the IUCN (International Union for Conservation of Nature) Red List [40].

#### Data analysis

Qualitatively, the data obtained were analyzed using the emic/etic approach [41]. This approach addresses the way in which the members of the studied culture perceive, structure, classify, and articulate their traditional knowledge. The “union of all individual competencies” model was also applied [42]; according to this model, all information pertinent to the investigated subject should be considered, thus structuring the ethnographic component of the study. The confirmation of the information provided by the interviewees occurred mainly in a synchronous manner, which consists of identical questions asked to different individuals in very short times [43]. Quantitatively, the data obtained were analyzed using the use value (UV), adapted from Phillips et al. [44] by Rossato et al. [45], which was used to determine the relative importance of each species as a function of each use and was calculated as UV = Σ*U*/*n*, where UV is the value of a species, *U* is the number of citations per species, and *n* is the total number of respondents/informants. The UV of each species is based only on the importance attributed by the interviewees and does not depend on any evaluation by the researcher [21, 46]. To estimate the richness of the traded species, an incidence matrix consisting of interviewees (rows) × type of species (columns) was prepared, assigning a value of 1 for each species mentioned by a respondent and 0 for those not mentioned. The species accumulation curves, in which the *X* axis corresponds to the number of individuals interviewed and the *Y* axis corresponds to the number of species used, were randomized 1000 times, and the mean values were calculated using the EstimateS software (version 8.2) [47]. The estimators Chao 1 and Jackknife 1 were used, which allow estimating the total number of species in a given area based on the sampled data.

Statistical analyses were performed between the socioeconomic factors (age, income, and education level) and the richness of species used by the interviewees. To assess the relationship between age and education level and the number of species used, Spearman’s correlations were performed for non-parametric data. To determine whether income influenced species richness, a Kruskal-Wallis test (*H*) was performed with the Dunn post hoc test. Spearman’s correlations analyses were also used to assess whether there was a relationship between the UV and the number of individuals (NI) of the cited species, the UV and the average purchase and sale values, the NI and the average purchase and sale values, and the purchase value and the profit obtained from the sale of songbirds. All these tests were performed using the software Paleontological Statistics—PAST 2.17c [48].

## Results

### Socioeconomic profile of the interviewees and richness and estimate of wild bird species traded

A total of 62 songbird keepers and/or traders were interviewed in the studied region (60 men and 2 women), with ages ranging from 11 to 78 years (mean of 34 years). Of the total respondents, 47 reported participating directly in the wild bird trade, while the others (*n* = 15) reported only using wild birds as pets because they like birds, in addition to appreciating their songs and company. More than half (*n* = 33) resided in rural areas, and 29 resided in urban areas; most of the respondents (*n* = 50) interviewed were originally from the studied region. Regarding education level, the levels reported by the interviewees were very low: 46 of the participants were illiterate or had only incomplete primary education.

In regard to the marital status of the interviewees, most were married (*n* = 26), followed by single (*n* = 24) and in a stable union (*n* = 8). The number of unemployed among the interviewees was very low (*n* = 6), and the occupations of the majority of respondents were agriculture (*n* = 21) and trade (*n* = 17). The income of the interviewees was low: most of them (*n* = 23) have a monthly income of US$ 499.04. There were no significant correlations between age (*p* = 0.868) or education level (*p* = 0.45) and the richness of species cited by the interviewees. The Kruskal-Wallis test showed that the number of species cited did not vary according to the income of the interviewees (*H* = 7.38; *p* = 0.111).

A total of 34 wild bird species belonging to 2 orders and 11 families were cited by the interviewees in the study region; the cited species originated from illegal trade and/or were captured by the interviewees themselves (Table 1). According to the interviewees’ testimonies, specific capture techniques are used for wild birds, which are targeted considering certain criteria such as the animal size, the best times of the year for the capture, and the feeding habit of different species. Respondents reported four capture techniques, the “assaprão,” “arapuca,” “visgo,” and “redinha or assaprão de rede,” with the assaprão being cited by most of the respondents (*n* = 35), followed by the visgo technique (*n* = 15). Detailed descriptions of these techniques can be consulted in [37].

**Table 1:** List of wild birds used for trade and as pets in the research area, including the scientific and popular names of each species, number of citations per use, use value (UV), and conservation status

Taxonomic categories (order/family/species)	Popular name	Citations per type of use		UV	Conservation status	
		Breeding	Trade		IUCN	MMA
Passeriformes						
Thraupidae						
*Sporophila lineola* (Linnaeus, 1758)	Lined seedeater	18	16	0.54	LC	LC
*Sporophila nigricollis* (Vieillot, 1823)	Yellow-bellied seedeater	28	21	0.79	LC	LC
*Sporophila ardesiaca* (Dubois, 1894)	Dubois’s seedeater	2	2	0.06	LC	LC
*Sporophila albogularis* (von Spix, 1825)	White-throated seedeater	30	22	0.83	LC	LC
*Sicalis luteola* (Sparrman, 1789)	Grassland yellow finch	4	3	0.11	LC	LC
*Volatinic jacarina* (Linnaeus, 1766)	Blue-black grassquit	7	6	0.20	LC	LC
*Sicalis flaveola* (Linnaeus, 1766)	Saffron finch	16	10	0.41	LC	LC
*Tangara cayana* (Linnaeus, 1766)	Burnished-buff tanager	5	5	0.16	LC	LC
*Tangara palmarum* (Wied, 1821)	Palm tanager	4	4	0.12	LC	LC
*Tangara sayaca* (Linnaeus, 1766)	Sayaca tanager	20	17	0.59	LC	LC
*Paroaria dominicana* (Linnaeus, 1758)	Red-cowled cardinal	27	24	0.82	LC	LC
*Sporophila angolensis* (Linnaeus, 1766)	Chestnut-bellied seed finch	1	1	0.03	LC	LC
*Sporophila bouvreuil* (Statius Muller, 1776)	Copper seedeater	4	0	0.06	LC	LC
*Coryphospingus pileatus* (Wied, 1821)	Grey pileated finch	3	1	0.06	LC	LC
*Saltator similis* d’Orbigny & Lafresnaye, 1837	Green-winged saltator	2	2	0.06	LC	LC
*Sporophila leucoptera* (Vieillot, 1817)	White-bellied seedeater	1	1	0.03	LC	LC
*Coereba flaveola* (Linnaeus, 1758)	Bananaquit	1	1	0.03	LC	LC
Icteridae						
*Icterus pyrrhopterus* (Vieillot, 1819)	Variable oriole	4	2	0.09	LC	LC
*Icterus jamacaii* (Gmelin, 1788)	Campo troupial	5	4	0.14	LC	LC
*Gnorimopsar chopi* (Vieillot, 1819)	Chopi blackbird	2	2	0.06	LC	LC
*Chrysomus ruficapillus* (Vieillot, 1819)	Chestnut-capped blackbird	1	0	0.01	LC	LC
Fringilidae						
*Spinus yarrellii* (Audubon, 1839)	Yellow-faced siskin	7	6	0.21	VU	VU B2ab(v)
*Euphonia chlorotica* (Linnaeus, 1766)	Purple-throated euphonia	3	1	0.06	LC	LC
Turdidae						
*Turdus rufiventris* Vieillot, 1818	Rufous-bellied thrush	15	14	0.46	LC	LC
*Turdus amaurochalinus* Cabanis, 1850	Creamy-bellied thrush	1	1	0.03	LC	LC
*Turdus leucomelas* Vieillot, 1818	Pale-breasted thrush	14	12	0.41	LC	LC
Mimidae						
*Mimus saturninus* (Lichtenstein, 1823)	Chalk-browed mockingbird	4	4	0.12	LC	LC
Cardinalidae						
*Cyanoloxia brissonii* (Lichtenstein, 1823)	Ultramarine grosbeak	23	22	0.72	LC	LC
Passerelidae						
*Zonotrichia capensis* (Statius Muller, 1776)	Rufous-collared sparrow	26	21	0.75	LC	LC
Corvidae						
*Cyanocorax cyanopogon* (Wied, 1821)	White-naped jay	1	1	0.03	LC	LC
Tyrannidae						
*Pitangus sulphuratus* (Linnaeus, 1766)	Great kiskadee	2	2	0.06	LC	LC
Estrildidae						
*Estrilda astrild* (Linnaeus, 1758)	Common waxbill	2	1	0.04	LC	LC
Psittaciformes						
Psitacidae						
*Eupsittula cactorum* (Kuhl, 1820)	Caatinga parakeet	2	1	0.04	LC	LC
*Amazona aestiva* (Linnaeus, 1758)	Turquoise-fronted amazon	1	1	0.03	LC	LC

**Fig. 2:**
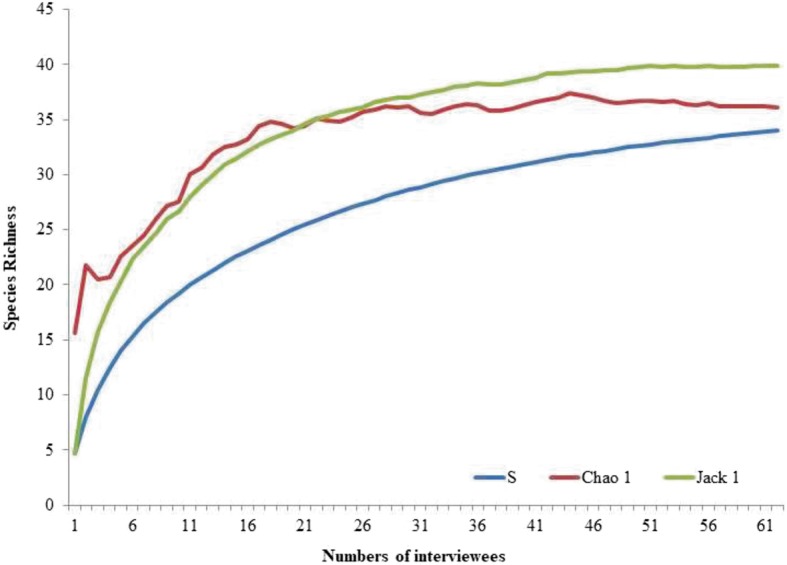
Rarefaction curves comparing the number of bird species observed (Sobs) with species richness estimated in the region studied (Chao 1 and Jackknife 1); calculated with 100 randomizations

The species richness (*n* = 34) recorded in the interviews was close to that projected by the Chao 1 (36 species, 94.4%) and Jackknife 1 (39 species, 87.2%) estimators, suggesting a sufficient sample size relative to the number of interviews (Fig. 2).

### Trade structure: routes and dynamics of the trade chain

A total of 47 respondents reported participating directly in the wild bird trade. According to the interviewees, the trade of songbirds occurs both among local keepers and traders and with those from other neighboring cities. That indicates that a high number of people are involved in these activities at the state level, forming a local and regional chain. Based on the interviews, illegal bird trade occurs mainly through the purchase and sale among those involved but also at points of sale such as bird street markets, at which birds are acquired at relatively low prices because, in most cases, they are young and/or “wild” adults (not trained in captivity). In the city where the present study was conducted, there is no wild bird trade in street markets; however, some interviewees indicated the da Prata and Central street markets as points of sale of birds, both located in the city of Campina Grande, located 11.36 km from the surveyed area.

Fowlers, known in Brazil as *passarinheiros*, i.e., those involved in the breeding and trade of wild birds in the study area, take on different roles in the trade chain: (1) catcher—this person captures the bird in the wild and sells it; in this case, the bird can be sold soon after capture or kept for a certain period of time in cages while being trained to develop their singing ability; (2) a reseller—this person is responsible for the purchase and sale of birds, usually buys an already trained bird from other resellers, and keeps the birds for a short time; and (3) end consumer—this person participates in the trade only for the purchase of birds to keep them as pets; in this study, consumers are also defined as keepers.

**Fig. 3:**
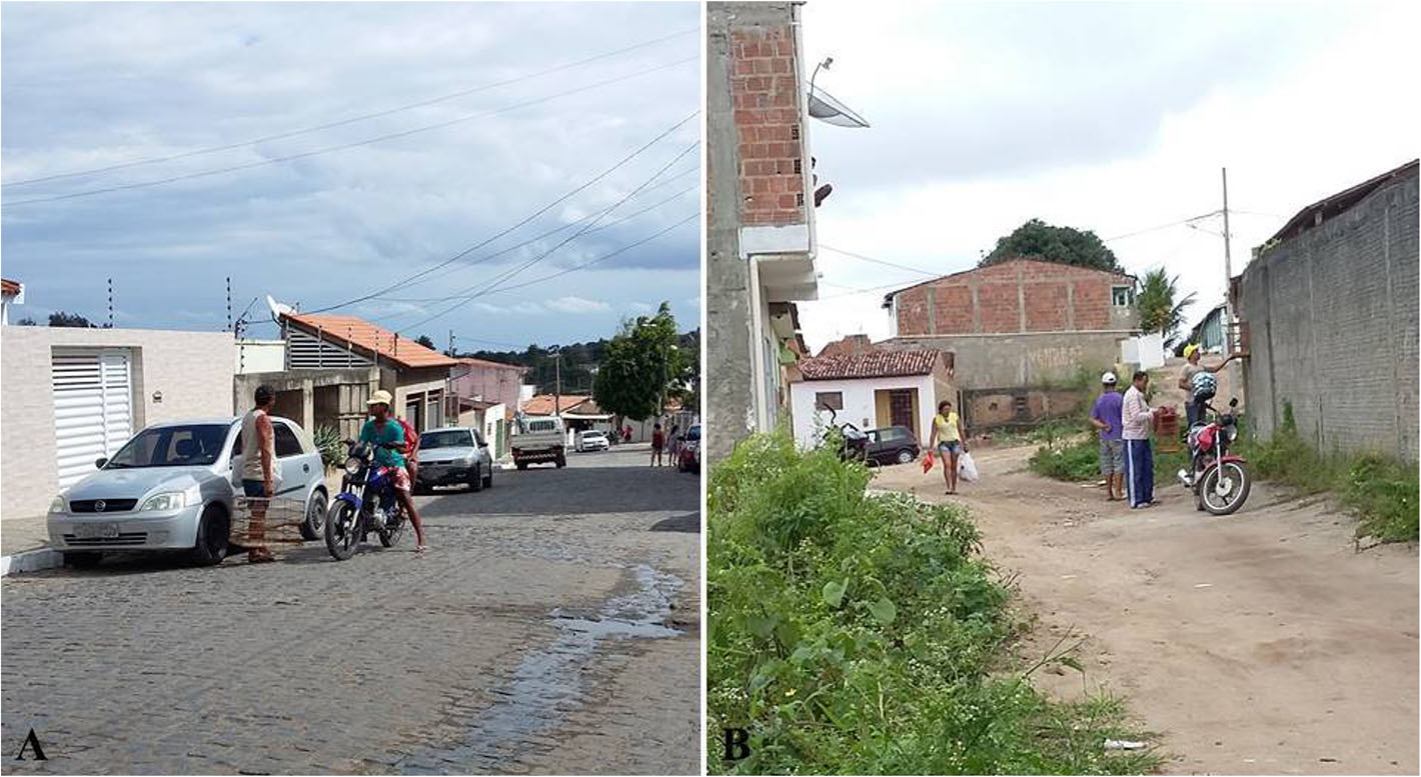
Wild bird trade among local keepers and traders. Interviewee trading a bird with other keepers and traders at a certain strategic point (**a**); interviewee trading a bird with another keeper and trader in front of his residence (**b**). Photos: Wallisson Sylas Luna de Oliveira

The purchase and sale dynamics in the trade chain reported in this study occur mainly between local keepers and traders. Trade occurs in the streets or in the homes of the interviewees, where urban traders travel to rural areas to buy or sell birds; the same occurs with traders from rural areas who travel to urban areas with the same purpose. Many interviewees reported traveling to neighboring cities to trade birds with keepers and traders from those cities as well as receiving visitors from other cities in their homes or at strategic locations to trade birds (Fig. 3).

**Fig. 4:**
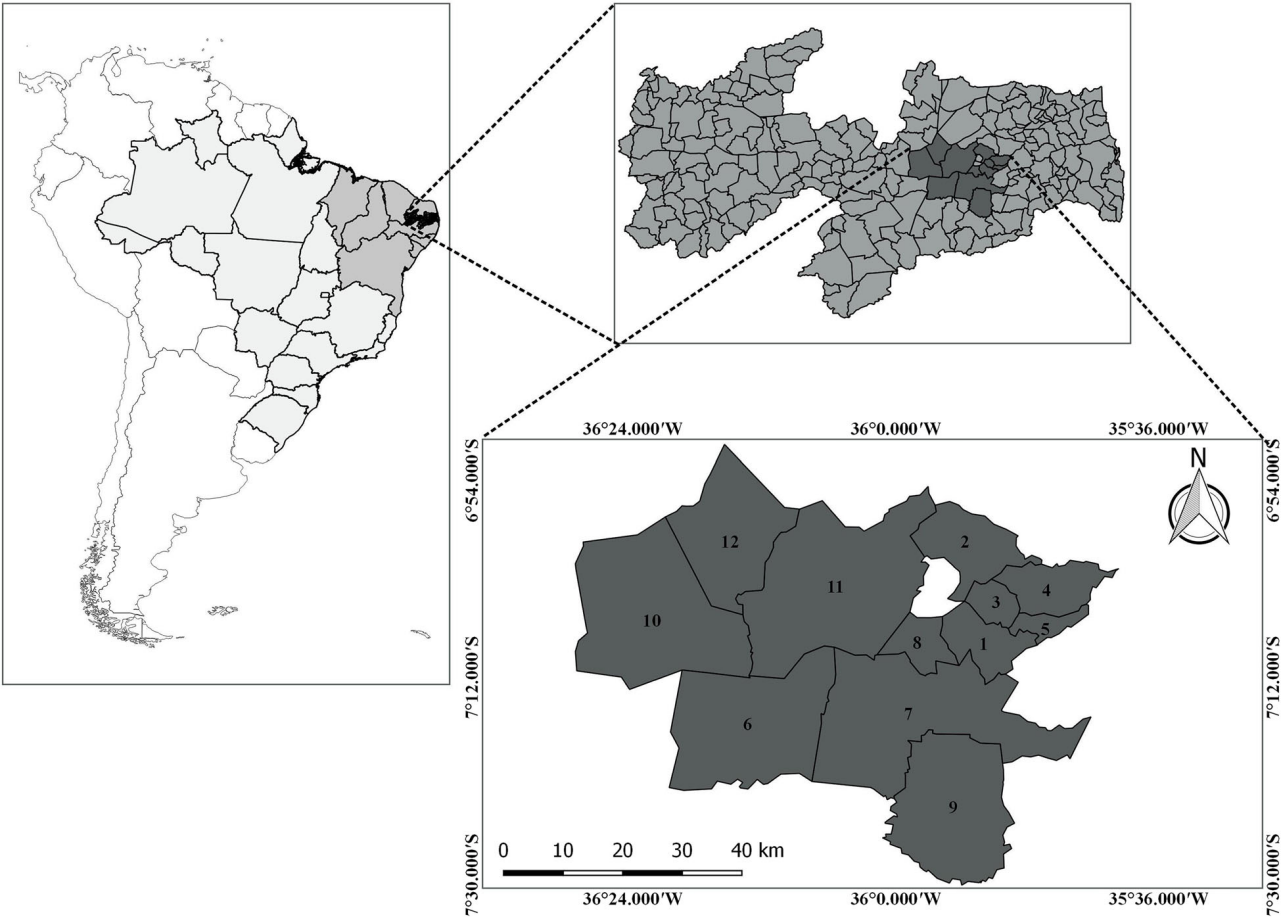
Area surveyed and nearby cities that make up the trade route in the region. (1) Lagoa Seca, (2) Esperança, (3) São Sebastião de Lagoa de Roça, (4) Alagoa Nova, (5) Matinhas, (6) Boa Vista, (7) Campina Grande, (8) Puxinanã, (9) Queimadas, (10) Soledade, (11) Pocinhos, and (12) Olivedos

Commercial routes include cities close to the surveyed area (Fig. 4). The identification of wild bird keepers and traders in these cities is facilitated by the geographical characteristics of the semiarid region, as the cities in this region are small and medium sized and connected by highways, enabling people involved in the trade chain to know each other. Although we have shown some limitations for the collection of information (because the activity is carried out clandestinely), the trade in this local and regional chain occurs daily but with greater intensity on weekends because according to the interviewees, it is safer then due to the lack of inspection by environmental agencies in cities and on highways and because most of the people involved in this chain work during the week. According to the interviewees, contact between those involved in local and regional trade is maintained via mobile phones, and in some cases, younger individuals use the WhatsApp messaging application. Additionally, according to the interviewees, this contact is important because certain birds can be ordered; thus, they schedule the day, time, and place to conduct trades/sales. One of the interviewees reported bringing birds from nearby cities when an order was made. According to him, these birds are dropped off in nurseries and kept as pets until their singing ability is good enough for trading. The use of personal motorcycles is very common among people involved in the trade chain; however, buses, private cars, and motorcycle taxis are also used to transport the animals that are traded between the cities involved or locally.

### Motives and factors that promote the local trade of wild birds

The availability, easy maintenance in captivity, ease of capture, and access to trade networks are factors driving the preference and choice of bird species that are traded and used as pets in the study area.

None of the interviewees reported that the trading of songbirds represents their main source of income. A total of 30 respondents reported trading birds as a form of “entertainment,” while 23 respondents said “extra income” is the main reason for selling songbirds.

### From song to profit: prices and profitability in local trade

In the area studied, keepers and traders classify the songs of various species according to their vocalization. The act of categorizing bird songs becomes an important factor as a criterion for choosing birds among keepers and traders because it facilitates the trade process among the people involved in these activities so that the average price of a particular bird is associated with its song. Questioned about which song characteristic adds value to a given species, many interviewees mentioned some names of songs, which according to them would be related to the sale value of the animal.

When songbirds are kept in captivity for a long period, they may develop the ability to sing other types of songs, either as a result of training or because they mimic and become accustomed to the songs of other birds. Among the songs of the birds reported in this study, the “wild” type stands out among the species cited. According to the interviewees, this type of song is recognized as the natural song of the bird in its environment and, in some cases, is related to species with high commercial value, as is the case of the rufous-bellied thrush *Turdus rufiventris* Vieillot, 1818, and the sayaca tanager *Tangara sayaca* (Linnaeus, 1766), which can reach values of up to US$ 229.88 and US$ 86.20, respectively. However, other species with a “wild” type song, such as the purple-throated *Euphonia chlorotica* (Linnaeus, 1766), the white-naped jay *Cyanocorax cyanopogon* (Wied, 1821), and the burnished-buff tanager *Tangara cayana* (Linnaeus, 1766), were sold for much lower prices, indicating that other factors, in addition to the songs, influence the price of birds.

**Fig. 5:**
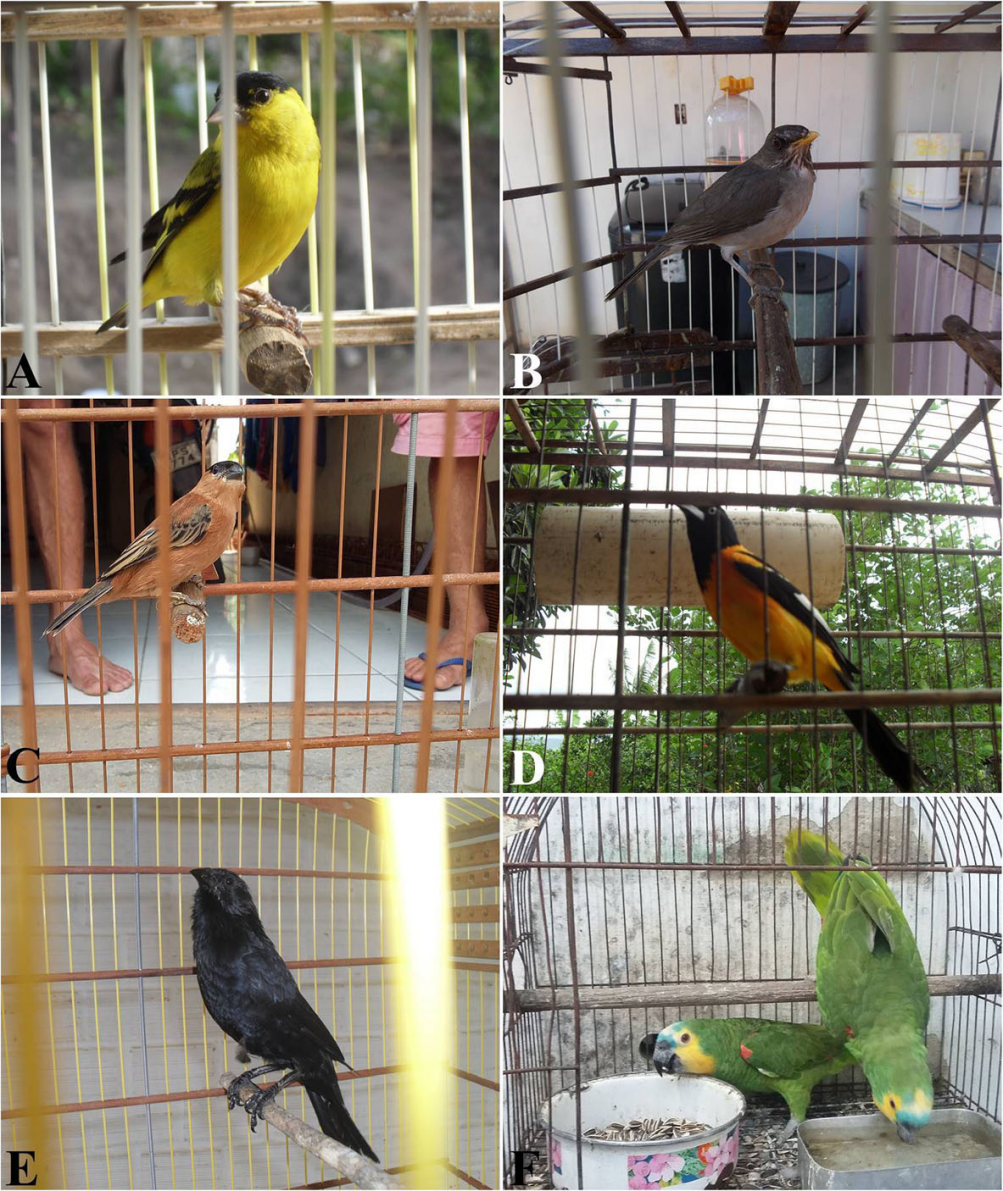
Record of species considered rare in the study area that were found in the interviewees’ homes. **a***Spinus yarrellii*. **b***Turdus amaurochalinus*. **c***Sporophila bouvreuil*. **d***Icterus jamacaii*. **e***Gnorimopsar chopi*. **f***Amazona aestiva*. Photos: Wallisson Sylas Luna de Oliveira

High prices were observed for some species with smaller numbers of individuals cited, namely, the yellow-faced siskin *Spinus yarrellii* (Audubon, 1839); the Dubois’s seedeater *Sporophila ardesiaca* (Dubois, 1894); the creamy-bellied thrush *Turdus amaurochalinus* Cabanis, 1850; the copper seedeater *Sporophila bouvreuil* (Statius Müller, 1776); the campo troupial *Icterus jamacaii* (Gmelin, 1788); the green-winged saltator *Saltator similis* (d’Orbigny & Lafresnaye, 1837); the chopi blackbird *Gnorimopsar chopi* (Vieillot, 1819); the turquoise-fronted amazon *Amazona aestiva* (Linnaeus, 1758); and the chalk-browed mockingbird *Mimus saturninus* (Lichtenstein, 1823), showing that the high prices in the trade chain may also be associated with both rarity in nature and in local trade (Fig. 5).

The importance of birds in the trade chain (inferred based on the UV) was found to be positively correlated (*p* < 0.05) with the NI traded and the average purchase and sale values of birds (Table 2). There was a strong correlation between the high UV of a given species and the number of traded individuals (*r*_s_ = 0.90; *p* < 0.001). Regarding the commercial potential of the species observed, it was found that birds with high UVs and high numbers of traded individuals also had higher average purchase and, especially, sale values (*r*_s_ = 0.52). This was observed especially for the red-cowled cardinal *Paroaria dominicana* (Linnaeus, 1758), the rufous-collared sparrow *Zonotrichia capensis* (Statius Muller, 1776), and the ultramarine grosbeak *Cyanoloxia brissonii* (Lichtenstein, 1823) (Table 3).

**Table 2:** Spearman’s correlation matrix demonstrating relationships between the variables use value (UV), number of individuals (NI), average purchase value (Purchase), and average sale value (Sale) of birds. The values in bold represent the correlation coefficient for each set of variables, and the other values represent the *p* value of the correlation

	UV	NI	Purchase	Sale
UV		0.001	0.028	0.002
NI	**0.90**		0.043	0.002
Purchase	**0.38**	**0.35**		0.001
Sale	**0.52**	**0.52**	**0.60**

**Table 3:** Price estimates reported by the interviewees for songbirds traded in the study region

Species	UV	NI	Average purchase value (US$)	Purchase value (US$)		Average sale value (US$)	Sale value (US$)	
				Minimum	Maximum		Minimum	Maximum
*Sporophila lineola*	0.54	24	29.52	2.87	114.94	37.50	7.18	100.57
*Spinus yarrellii*	0.21	11	14.36	14.36	14.36	43.10	14.36	100.57
*Sporophila nigricollis*	0.79	39	26.87	2.01	114.94	27.55	6.32	71.83
*Sporophila ardesiaca*	0.06	2	100.57	100.57	100.57	0.00		0.00
*Sporophila albogularis*	0.83	39	21.33	1.29	86.20	22.32	7.18	114.94
*Zonotrichia capensis*	0.75	45	73.92	8.62	172.41	50.43	8.62	143.67
*Sicalis luteola*	0.11	5	26.86		25.86	22.98		22.98
*Euphonia chlorotica*	0.06	9	1.46		1.46	2.87		2.87
*Estrilda astrild*	0.04	4	0.00		0.00	7.18		7.18
*Volatinia jacarina*	0.21	19	8.62		8.62	22.98	14.36	31.60
*Turdus rufiventris* Vieillot, 1818	0.46	21	67.36	14.36	244.25	39.91	25.86	57.47
*Turdus amaurochalinus* Cabanis, 1850	0.03	1	43.10		43.10	0.00		0.00
*Turdus leucomelas* Vieillot, 1818	0.41	21	20.11	14.36	28.73	30.45	17.24	43.10
*Cacicus cela*	0.09	4	5.74	4.31	8.62	11.49	8.62	14.36
*Sicalis flaveola*	0.41	40	27.03	7.18	57.47	26.26	17.24	57.47
*Cyanoloxia brissonii*	0.72	36	48.22	25.00	373.56	69.48	8.62	229.88
*Tangara cayana*	0.16	9	2.29		2.29	11.49		11.49
*Tangara palmarum*	0.12	3	0.00		0.00	20.11		20.11
*Tangara sayaca*	0.59	27	29.45	4.31	43.10	40.22	14.36	86.20
*Paroaria dominicana*	0.82	40	73.16	2.29	344.82	52.67	14.36	143.67
*Cyanocorax cyanopogon*	0.03	1	0.00		0.00	8.62		8.62
*Sporophila bouvreuil*	0.06	4	57.47		57.47	7.18		7.18
*Coryphospingus pileatus*	0.06	1	0.00	57.47	0.00	0.00		0.00
*Icterus jamacaii*	0.14	5	29.09	7.18	71.83	50.28	28.73	71.83
*Pitangus sulphuratus*	0.06	6	0.00		0.00	43.10		43.10
*Saltator similis* d’Orbigny & Lafresnaye, 1837	0.06	2	122.12	43.10	201.14	140.80	51.72	229.88
*Gnorimopsar chopi*	0.06	3	114.94	114.94	114.94	114.94		114.94
*Amazona aestiva*	0.03	4	83.33	80.45	86.20	114.94		114.94
*Mimus saturninus*	0.12	5	24.42	20.11	28.73	64.65	28.73	350.00
*Eupsittula cactorum*	0.04	5	8.62		8.62	11.49		11.49
*Coereba flaveola*	0.03	1	8.62		8.62	20.11		20.11

In the present study, the prices of birds traded by the interviewees ranged from US$ 1.29 to US$ 373.56 (Table 3). The high purchase and sale values of songbirds (either individually or due to the large volume of specimens traded) show the importance of a significant economic return for those involved in this activity.

The price variation for songbirds reported in this study is also a result of the way in which the specimens are acquired. Birds newly caught from the wild are sold at very low prices because they are considered “wild” (they acquire that name because they have not yet gone through the adaptation period in captivity) and so are, in most cases, young birds whose songs are still not attractive for consumers. On the other hand, when kept in captivity for a long period of time after capture, after training until reaching adulthood and developing singing abilities, birds are added by fowlers to the trade chain at higher commercial values, becoming a profitable activity for those involved.

A total of 21 respondents reported their preference for capturing birds directly from nature to keep them in captivity until adulthood and then sell them to keepers and traders. According to these interviewees, this trade procedure is more profitable because they do not invest money in the acquisition of these birds and add value to the specimen during the training period in captivity. However, considering that the maintenance of songbirds in captivity for a long time requires a certain financial expense with food and, in some cases, with medicines and vitamins for the birds, in the long term, the profit with the trade of these birds is not as significant as the profitability with birds acquired from trade.

**Fig. 6:**
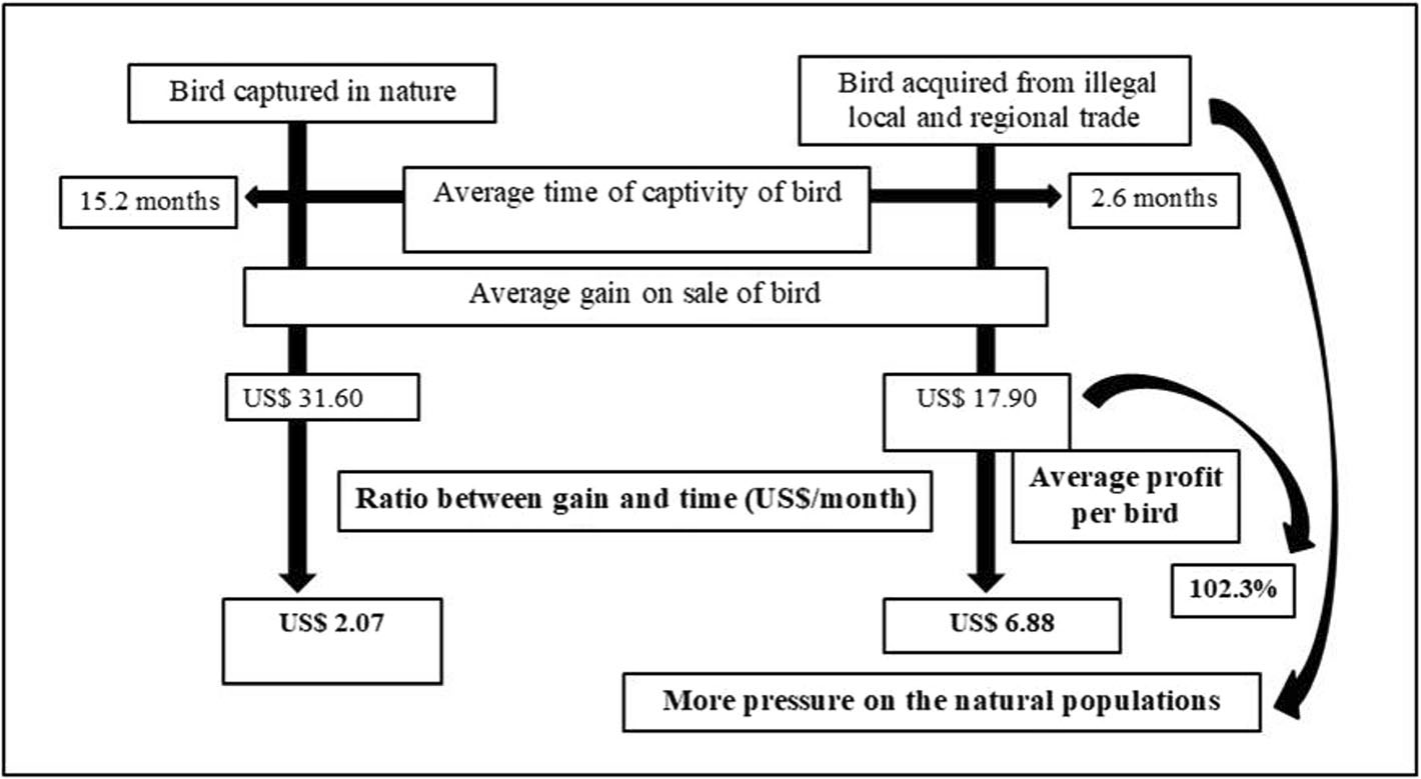
Songbird illegal trade chain in the semiarid region of Paraíba

The average time of captivity of these birds was used to estimate the difference in gain and profitability between the sale of songbirds acquired through capture in the wild and of birds acquired from trade (Fig. 6). When comparing the average time of captivity of birds captured in nature and birds acquired from illegal trade, a significant difference of 15.2 and 2.6 months per bird was observed, respectively, generating an average gain per bird of US$ 31.60 for birds captured in nature and US$ 17.90 for birds acquired from local and regional illegal trade. However, a bird acquired through illegal trade generates a profit of US$ 6.88/month, and a specimen captured in nature and kept for some time in captivity by the trader generates only US$ 2.07/month (Fig. 6).

**Fig. 7:**
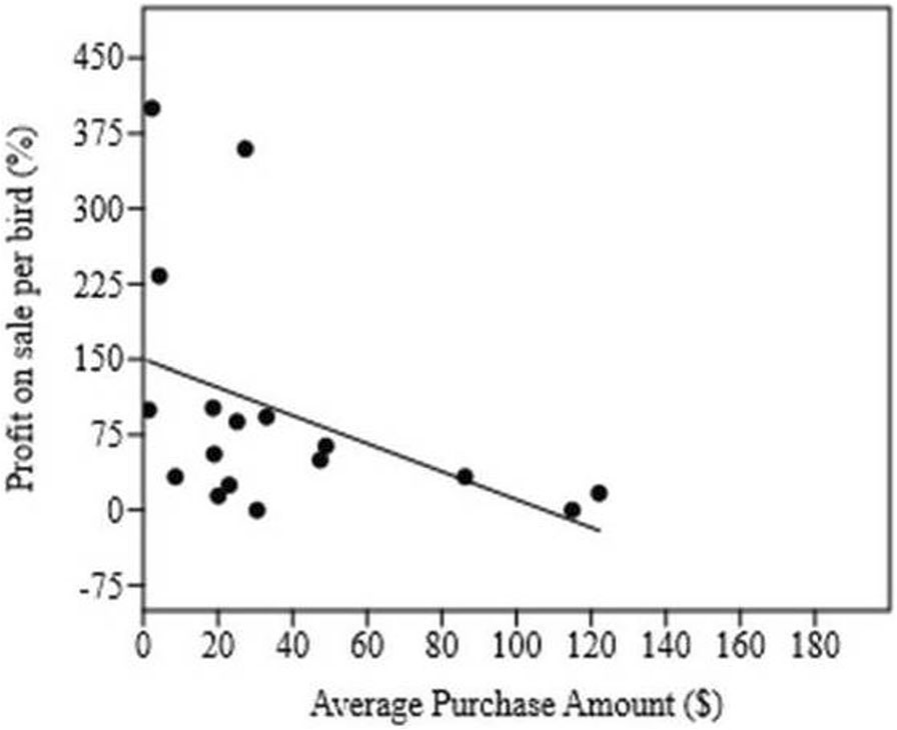
Correlation of profit and average purchase value for illegal songbird trade in the Lagoa Seca municipality, Paraíba

In addition to the shorter period of captivity, the purchase price of birds is another determining factor in obtaining quick profits in the trade chain. Birds with lower purchase values have higher sale profits (*r*_s_ = − 0.54; *p* = 0.02) (Fig. 7). Thus, while more expensive birds are sold a few times in a year, generating low profits, others of lower values are traded often, generating a continuous income and a much higher profit due to the greater number of individuals sold.

**Fig. 8:**
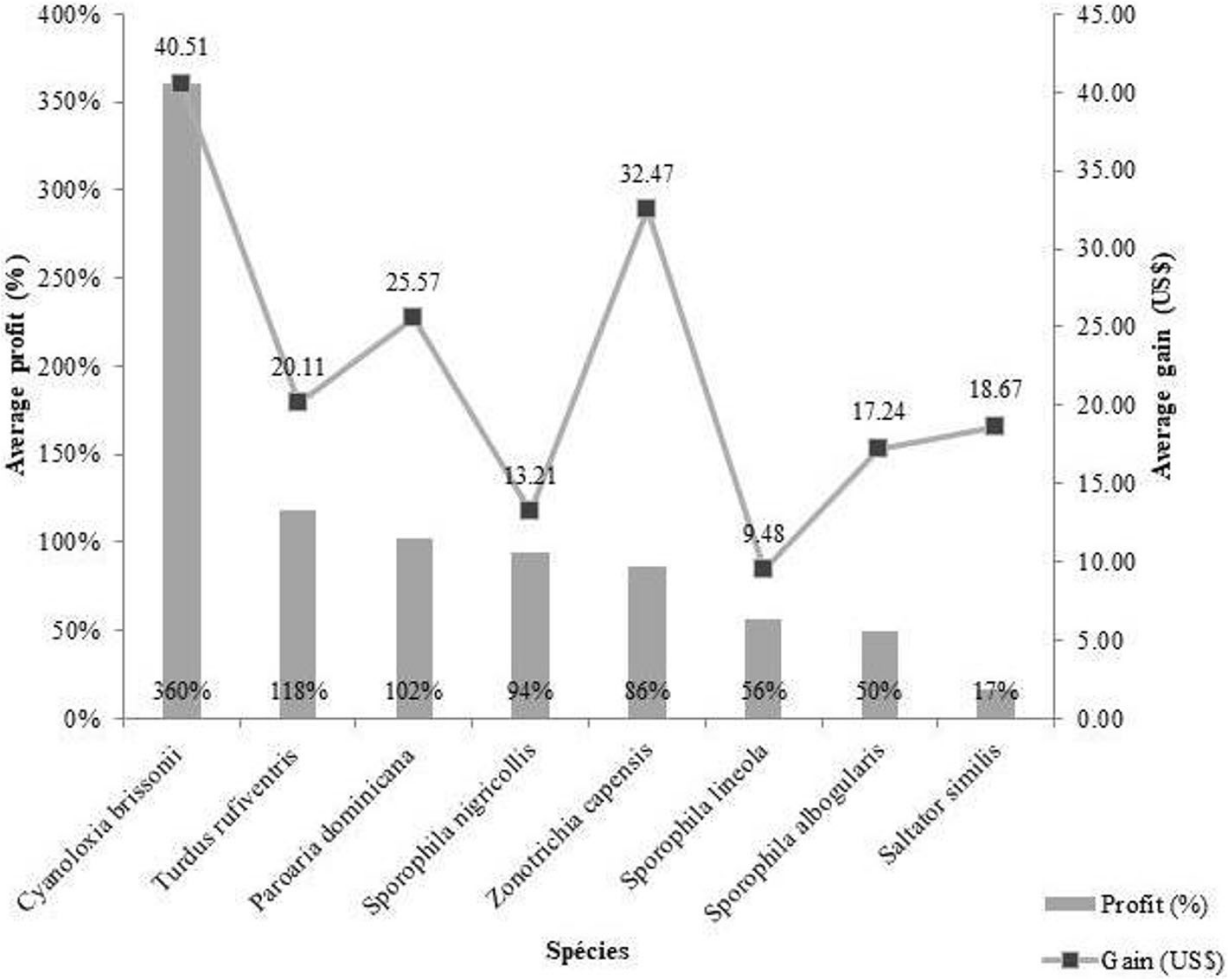
Average profit and gain among songbird species traded by the interviewees in the present study (average gain (US$) = sale value − purchase value; average profit (%) = gain value/purchase value × 100)

Among the species reported in the present study, those that showed individuals with higher profit percentages included the ultramarine grosbeak *C. brissonii*, *T. rufiventris*, *P. dominicana*, and *Sporophila nigricollis* (Vieillot, 1823) (Fig. 8); however, these species also presented individuals relatively expensive in terms of purchase prices.

## Discussion

The illegal sale of songbirds in the study area reflects a history of strong interaction among residents of the semiarid Northeast Region, where wild birds are traditionally kept as pets, a practice that persists in the region despite the legal implications. The richness of bird species recorded is not surprising, as these animals are often kept as pets and are chosen because of their diversity of colors, song, ease of maintenance, and, in some cases, ability to imitate the human voice [49, 50]. These factors have led to the preference of bird keepers for wild pets, especially Passeriformes [51–55] in the studied area, in other locations in Brazil, and worldwide. The main capture techniques mentioned by the interviewees in the researched area are widely used in other areas of the Brazilian semiarid [24, 26, 51] making it possible to capture a large number of species.

The organization of the trade chain in a decentralized manner in the area studied highlights a strategy of traders, who avoid trading at fixed points of sale (e.g., street markets and open-air markets) vulnerable to inspection. A similar situation was observed in a study conducted by [54] on the Passeriformes trade in Floriano, Piauí, Brazil, where most of the trade of these birds takes place outside of street markets and is often performed in the homes of traders or end consumers. Similar results were also reported in studies on the trade of herpetofauna species for use as pets in some regions of Thailand, where the trade pattern based on local markets [56] migrated completely to another venue essentially characterized by sales in traders’ homes [57].

The Campina Grande and Soledade municipalities, which were identified in the present study as places of sale of wild birds at street markets and by order, respectively, are regional and national trafficking centers from where a considerable amount of wild animals are trafficked for several regions in Brazil and abroad [16, 58, 59]. In addition, these 2 municipalities are crossed by federal highway BR-230, known for being an outlet of wild animals trafficked in the Brazilian semiarid, crossing the states of Piauí and Maranhão and interconnected to other highways that connect to Goiás and São Paulo [60]. In Brazil, the trafficking routes cross the country in the north-south direction via main highways, and the North, Northeast, and Central-West regions are primarily responsible for the high demand for wild animals illegally traded in Brazil [58, 61].

Given the decentralization of this trade chain, the ease of availability of species is one of the main factors driving the illegal trade of wild birds in areas of the Brazilian semiarid. This pattern has also been recorded in other studies on the trade of wild birds as pets in Latin America [3, 23, 26, 27, 59, 62–65], China [66], and Southeast Asia [67–70].

In addition to the sociocultural and socioeconomic aspects, factors related to the relationship between humans and birds, such as bird song attractiveness and singing ability, beauty of the plumage, the ability to imitate the human voice (e.g., psittacine), and companionship [71–74], are crucial for promoting the trade of several bird species among the people involved. Previous studies have demonstrated that wild bird trade activities are very profitable in the context of high unemployment rates and low levels of formal education in Brazil and a very large and growing business at the global scale [3, 75].

In the study area, the purchase or sale values of songbirds are directly associated with the attractiveness and type of song of a particular bird. This situation reflects the importance of vocalization as an attribute of birds involved in the pet trade in Brazil [3, 25, 27, 54] and in other countries. An example is a case study of individuals of the species *Copsychus malabaricus* conducted in Medan, Indonesia, which compared the preference between wild songs and the songs of birds bred in captivity [76]; many bird owners preferred the song of birds captured in nature because the wild song of this species is considered superior to the song of individuals bred in captivity.

The increase in the price of birds based on the rarity of species in the study area has also been evidenced in previous studies. Souto et al. [54] indicated that rarity was the second most determinant factor in the prices of Passeriformes. In a study on factors that determine the price of pet wild birds sold in pet shops in Taiwan, Su et al. [77] observed that rare species, i.e., less abundant species, had a higher commercial value, while more abundant species were less expensive. In addition, the authors noted other factors related to bird prices, such as body size, beauty of the plumage, and song.

The way bird specimens are acquired, as well as the acquisition preference of traders, has been another important factor in determining profitability from the sale of these animals. Our data corroborate the results of Fernandes-Ferreira et al. [25] in a study on songbird trade in the state of Ceará, Brazil, where it was found that the value of a red-cowled cardinal *P. dominicana* ranged from US$ 1.72 for newly captured individuals to US$ 172.41 for an excellent singer reared in captivity. The red-cowled cardinal *P. dominicana* is among the species with the highest UVs in the present study and has also been highlighted as one of the most traded birds in other areas of northeast Brazil [20, 23, 25, 27, 51, 53, 78].

Other previous studies suggest that the abundance and popularity of species of the genus *Sporophila* are factors that make them the most frequently recorded in studies on the breeding and trade of songbirds in northeast Brazil [20, 23, 25–27, 49, 51, 53, 54]. Pagano et al. [79] recorded the preference for birds of the genus *Sporophila* traded in street markets in the state of Paraíba, noting that the low prices of the birds of this genus have contributed to their popularity.

## Conclusions

The songbird trade in the semiarid region of Brazil involves keepers and traders from various cities. Trade occurs mainly through the purchase and sale between local keepers and traders, and the expansion of this trade to people from other neighboring cities is common. The proximity between the cities that form the trade routes, the ease of availability of the means of communication used in commercial transactions, and the use of personal transportation for the movement of traders between cities are factors that favor and promote the illegal trade of songbirds among those involved. Although the results of this study demonstrate that the illegal trade of songbirds is not recognized as the main source of income for people involved in it, this activity represents an additional source of income. Given these situations, attention should be paid to the conservation aspects of bird species involved in the trade chain, especially those reported in greater numbers in the present study because those species are strongly impacted by exploitation.

The purchase and sale values of birds are influenced by factors that deserve attention both from socioeconomic aspects of those involved in this activity and from conservation aspects of the species involved in the trade chain. The value of traded birds is associated with the rarity or abundance of some species and especially the type of song and singing ability of a particular bird, regardless of species. In addition, the knowledge of the interviewees regarding vocalizations and the types of songs produced by birds is a factor of great importance in determining the purchase and sale values of birds.

There is a significant difference in profit between the sale of birds captured from nature and of birds acquired directly from trade. Considering the period in captivity of birds in these 2 situations, birds acquired directly from trade generate higher average profits in the trade chain. In addition, the results of this study also showed that birds with lower purchase values generate higher profitability, as the affordable price of these birds attracts a larger number of people from all social classes, leading to a relatively large demand for individuals of several species, especially those of the genus *Sporophila*, which includes a greater number of individuals involved in the trade chain in the semiarid region of Paraíba.

Given the above, our results suggest that more effective control actions in the fight against environmental crimes should be implemented (Law 9.605/98) so that inspection agencies can intensify periodic monitoring on the main roads and highways, including on weekends, as a way to minimize the impacts of the exploitation of wild birds caused by illegal capture and trade. In addition, environmental education programs through mass media campaigns and visual advertisements reporting the mistreatment and mortality rates of the species involved since capture, transport, and trade to their final destination can be important tools for raising awareness about the conservation of exploited wild bird species.

## Data Availability

All data generated or analyzed during this research are included in this published article

## Abbreviations

CAAE: Certificate of Presentation for Ethical Consideration
CBRO: Brazilian Ornithological Records Committee
CETAS: Wild Animal Triage Centre
HDI: Human Development Index
HULW: Hospital Universitário Lauro Wanderley
IBAMA: Brazilian Institute of the Environment and Renewable Natural Resources
IUCN: International Union for Conservation of Nature
MMA: Ministry of the Environment
PAST: Paleontological Statistics
UV: Use value
